# Thermal perturbation of NMR properties in small polar and non-polar molecules

**DOI:** 10.1038/s41598-020-63174-6

**Published:** 2020-04-08

**Authors:** Nicholas R. Jaegers, Yong Wang, Jian Zhi Hu

**Affiliations:** 1 Institute for Integrated Catalysis and Earth and Biological Science Directorate, Pacific Northwest National Laboratory, Richland, Washington, 99354 United States; 2 Voiland School of Chemical Engineering and Bioengineering, Washington State University, Pullman, Washington, 99163 United States

**Keywords:** Catalysis, Chemistry, Analytical chemistry, NMR spectroscopy, Solid-state NMR

## Abstract

Water is an important constituent in an abundant number of chemical systems; however, its presence complicates the analysis of *in situ* ^1^H MAS NMR investigations due to water’s ease of solidification and vaporization, the large changes in mobility, affinity for hydrogen bonding interactions, etc., that are reflected by dramatic changes in temperature-dependent chemical shielding. To understand the evolution of the signatures of water and other small molecules in complex environments, this work explores the thermally-perturbed NMR properties of water in detail by *in situ* MAS NMR over a wide temperature range. Our results substantially extend the previously published temperature-dependent ^1^H and ^17^O chemical shifts, linewidths, and spin-lattice relaxation times over a much wider range of temperatures and with significantly enhanced thermal resolution. The following major results are obtained: Hydrogen bonding is clearly shown to weaken at elevated temperatures in both ^1^H and ^17^O spectra, reflected by an increase in chemical shielding. At low temperatures, transient tetrahedral domains of H-bonding networks are evidenced and the observation of the transition between solid ice and liquid is made with quantitative considerations to the phase change. The ^1^H chemical shift properties in other small polar and non-polar molecules have also been described over a range of temperatures, showing the dramatic effect hydrogen bonding perturbation on polar species. Gas phase species are observed and chemical exchange between gas and liquid phases is shown to play an important role on the observed NMR shifts. The results disclosed herein lay the foundation for a clear interpretation of complex systems during the increasingly popular *in situ* NMR characterization at elevated temperatures and pressures for studying chemical systems.

## Introduction

Water is a ubiquitous constituent in numerous chemical systems. Despite its vast importance in biological life and societal development, new discoveries in its physical and chemical properties are being made on an ongoing basis, highlighting the vast uncertainties this molecules offers^1^. Its presence in multi-component systems often introduces enhanced complexities in the behavior and interpretation of the chemical system. For example, water has been shown to both improve and retard the rates of C-H activation in zeolites while enhancing Fischer-Tropsch synthesis rates on ruthenium, illustrating water’s intricate interactions with catalytic materials^2,3^. Identifying the specific interactions of water with such materials can be complicated by the challenging interpretation of spectroscopic data collected for water on material surfaces. It poses particular challenges when interpreting *in situ* solid-state nuclear magnetic resonance (NMR) spectroscopy data, an increasingly prevalent spectroscopic technique owing to the presence of protons in most chemical systems, and its status as the most sensitive NMR nuclear spin (^1^H) enhances the temporal resolution possible for this technique. Spectroscopic interpretation challenges from water arise due to the high mobility of the molecule and its affinity for hydrogen bonding interactions which result in a dramatic temperature-dependent chemical shielding of the H_2_O molecule. Substituting H_2_O for D_2_O in complex systems can provide enhanced clarity in some instances, but H-D exchange may still be present which complicates analysis. The ability to understand and interpret the signatures of water under reactive environments has become increasingly important as *in situ* and *operando* NMR studies become more prominent. The non-destructive insight provided for applications such as geochemistry or catalysis reveals much about the chemical systems, where the structure of water in porous materials and on surfaces play an important role in reactivity and modifying the active centers^4–8^. In protonated MFI zeolite, for instance, water has been shown to interact with Brønsted acid sites to form hydrated hydronium ions which can serve as the catalytically active center^4^. To understand and interpret the signatures of water and other solvents in more complex systems at elevated temperatures, foundational work on these constituents alone over a range of conditions is of paramount importance. Previous efforts have investigated such a domain^9^; however, these studies were relatively limited in both temperature range and thermal resolution. Recent developments in the operational range of solid-state NMR have allowed for temperatures exceeding 200 °C and 200 bar, which enables an expansion of the range of reported NMR properties for water beyond what was previously known and provides a framework for employing each chemical shift, linewidth, and relaxation time as a gauge for the behavior of water^10,11^. Since systems containing water and other small molecules may well be analyzed at such harsh conditions, this extension of the scientific understanding for the spectroscopic behaviors of water will aid in the interpretation of cutting-edge *in situ* experimentation. Herein, the spectroscopic properties of such molecules are detailed and interpreted on the basis of physical phenomena to provide clarity in the behavior of waters in complex reaction systems. Specifically, the reported temperature range for NMR properties (^1^H and ^17^O chemical shift, linewidth, and spin-lattice relaxation time) was expanded beyond previous reports and links published discrete findings on the behavior of water to observable NMR properties. Such an approach has enabled the observation of tetrahedral patching through the ^1^H linewidth, a reduction in hydrogen bond strength at elevated temperatures, and the potential impact of gas to liquid chemical exchange on the observed NMR properties.

## Results & Discussion

**Figure 1:**
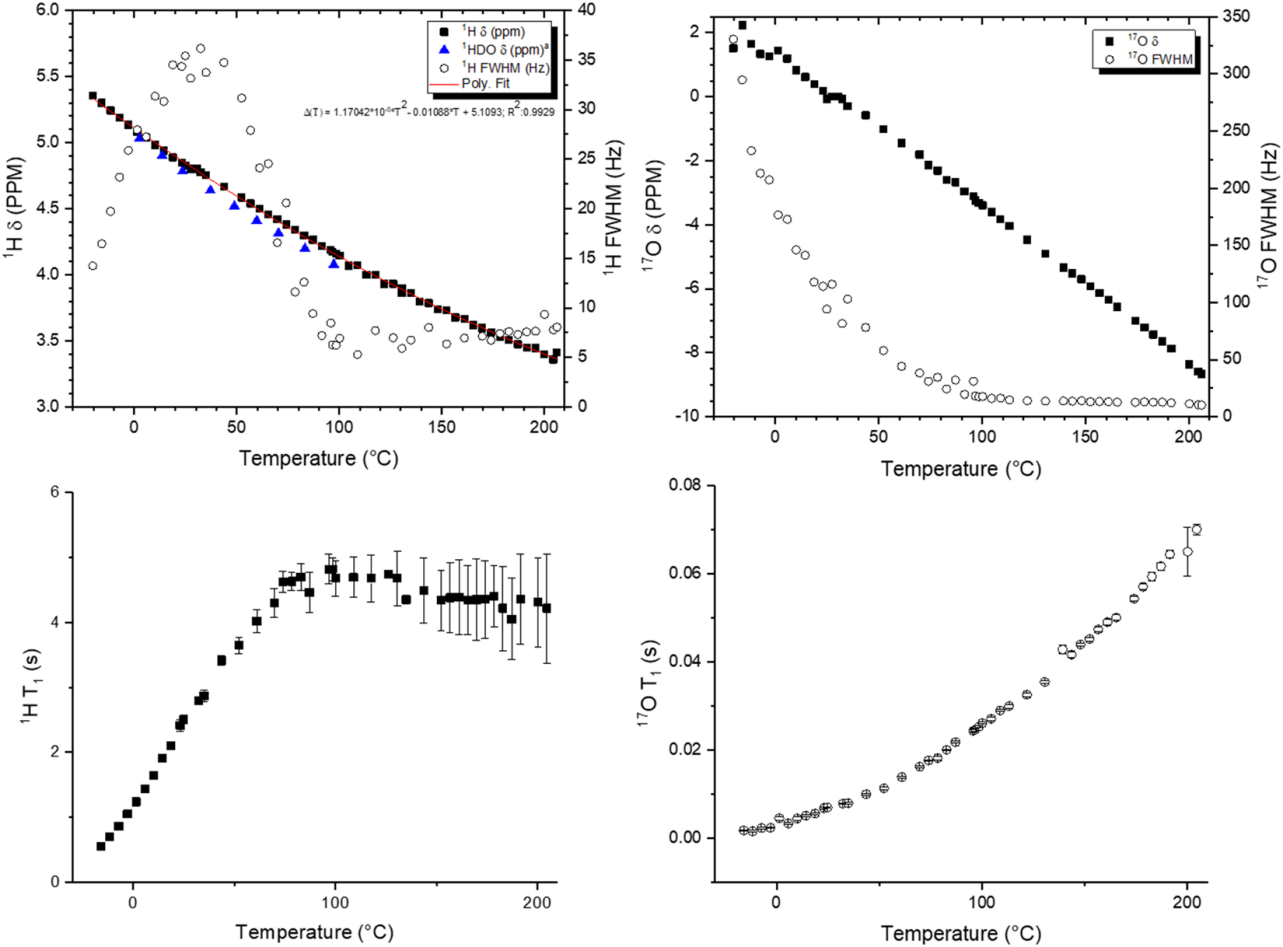
Proton (^1^H, left) and oxygen (^17^O, right) NMR data collected for chemical shift (filled) and full-width at the half-maximum (open) (top) and spin-lattice relaxation time (bottom) as a function of sample temperature. 200 μl of 20% H_2_^17^O was loaded into the rotor to maintain primarily condensed phase water throughout the experiments. ^a^Data extracted from Gottlieb *et al*. *J Org Chem* **1997**, 62, 7512–7515.

The effect of condensed-phase water (18.2 MΩ·cm) on the chemical shift, linewidth, and spin-lattice relaxation time of ^1^H and ^17^O species as a function of the state-of-the-art range of temperature for solid-state NMR are shown in Fig. 1. Though magic-angle spinning is not required for liquid-state NMR analysis, it will not impact the observations herein^12^ and will aid spectroscopy at super-cooled temperatures where the presence of solid water results in anisotropies in nuclear interactions (see below). The presented data illustrate that an increase in temperature results in a continual, non-linear decrease of the ^1^H chemical shift of water. This parameter was found to follow the relationship of $\varDelta (T)=1.17042\ast {10}^{-5}\ast {T}^{2}-0.01088\ast T+5.1093$ across the temperature range examined. This decrease in chemical shift can be explained by changes to the hydrogen bonding network of liquid water, which are perturbed by thermal energy and motions. As the temperature of water increases, hydrogen bonding interactions are suppressed as is often the case for hydrogen-bonded species^13,14^. In fact, the molecular dipole moment of water at high temperatures has been predicted to take a value intermediate of isolated gas phase molecules and ambient liquid, illustrating a consistent prediction of weakened hydrogen bonding behavior to that observed experimentally^15^. The weakening of hydrogen bonding results in stronger O-H covalent bonds and the stronger covalent interaction, in turn, increases electronic shielding of the proton nuclei since it is drawn closer to the electronic environment of the neighboring oxygen atom. This weakening of hydrogen bonding interactions directly manifests itself in the observed chemical shift of condensed phase water over the wide range of temperature displayed. This trend is consistent with previous data (extracted and plotted as blue triangles) for liquid water over a narrower range than presently available^9^. Observation of the ^1^H chemical shift at super-cooled temperatures was also possible in part due to the enhanced pressure inside the fluid as the rotor spins as well as the premelting phenomenon^16^. Similar to the observed ^1^H chemical shift trends, ^17^O chemical shifts decrease steadily over the wide temperature range, with greatly enhanced thermal resolution over what was previously reported^17^. The observed decrease in chemical shift is a direct consequence of the increase in diamagnetic shielding of as hydrogen bonding is weakened at higher temperatures.

**Figure 2:**
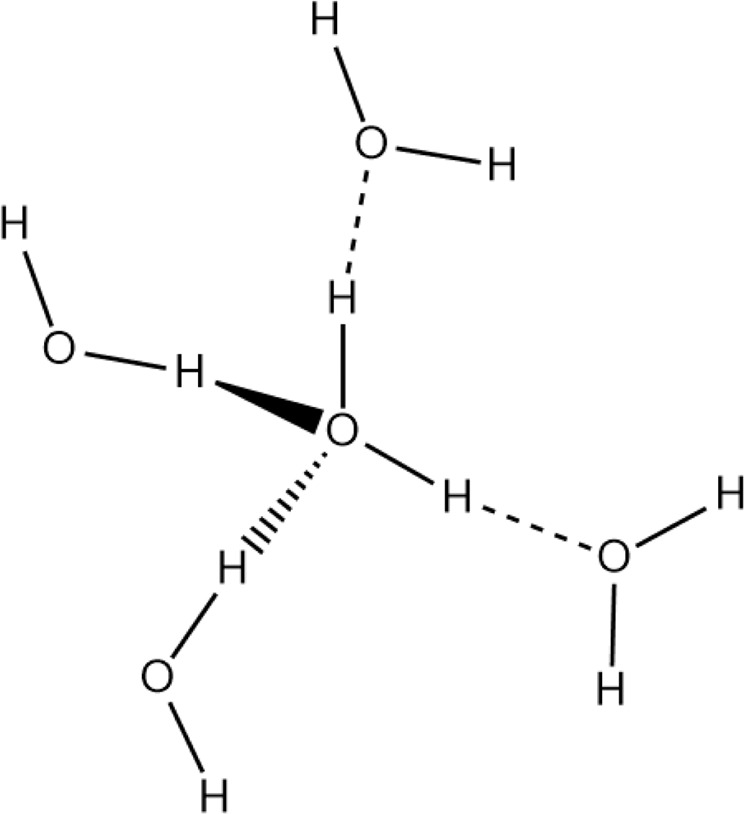
Representative scheme of tetrahedrally-coordinated water for illustrative purposes.

The linewidth profile of the ^1^H spectra follows a non-trivial profile across the wide temperature range presented. In the high temperature regime, fairly narrow lines are present due to the high mobility of water molecules when excited by thermal energy. This increased mobility reduces the variation in observed electronic environment due to signal averaging induced by the quick random molecular motion. As the temperature decreases below 100 °C, the linewidth increases due to a reduction in the mobility of water. At ~50 °C, however, the linewidth reaches a maximum and decreases at temperatures lower than this value. Smaller linewidths below this temperature may indicate an increase in ordering of the liquid water. Indeed, it has been noted that the onset of tetrahedrally-coordinated domains in water occurs around 50 °C^15,18^. This ordered structure, depicted in Fig. 2, likely accounts for the decrease in linewidth due to the uniformity of sites. At higher temperatures in this regime, the rigorous bonding network becomes increasingly distorted, which increases the linewidth. At temperatures above this regime, the linewidth is driven by mobility. This tradeoff between ordering at lower temperatures and enhanced random molecular motion at high temperatures results in a volcano-shaped linewidth profile at lower temperatures, serving as the first-known correlation between the tetrahedral ordering and mobility of water molecules and such a volcano-type linewidth profile. Such an observation offers linewidth as a method to better understand the properties and mobility of water in complex systems. It should be noted that the absolute values for linewidth will strongly depend on the instrument, shimming, chemical environment, and operational conditions, but the trends expressed herein should well-represent expectation. In contrast to ^1^H NMR, the linewidths for ^17^O spectra are about an order of magnitude larger than the ^1^H linewidths and continually decrease as the temperature increases up to 100 °C and the mobility of the water molecules increases. The larger nucleus and linewidth ^17^O atoms are less impacted by the ordering of water molecules in tetrahedral patches under these conditions.

Spin-lattice relaxation times were measured for each nucleus using an inversion-recovery pulse sequence. As the mobility of the proton species increased, the measured T_1_ also increased, explained by a reduction in correlation time as tetrahedral ordering is reduced and mobility dominates. Above 100 °C, the ^1^H T_1_ measurement leveled off at about 4.5 s. The increased error in this high-temperature regime is a consequence of longer relaxation times and insufficient recycle delay. In contrast to the proton T_1_ measurements, ^17^O spin-lattice relaxation times continually increased over the reported temperature range due to a decrease in correlation time. It should be noted that the absolute values for T_1_ are dependent on the magnetic field, but the trends are representative.

**Figure 3:**
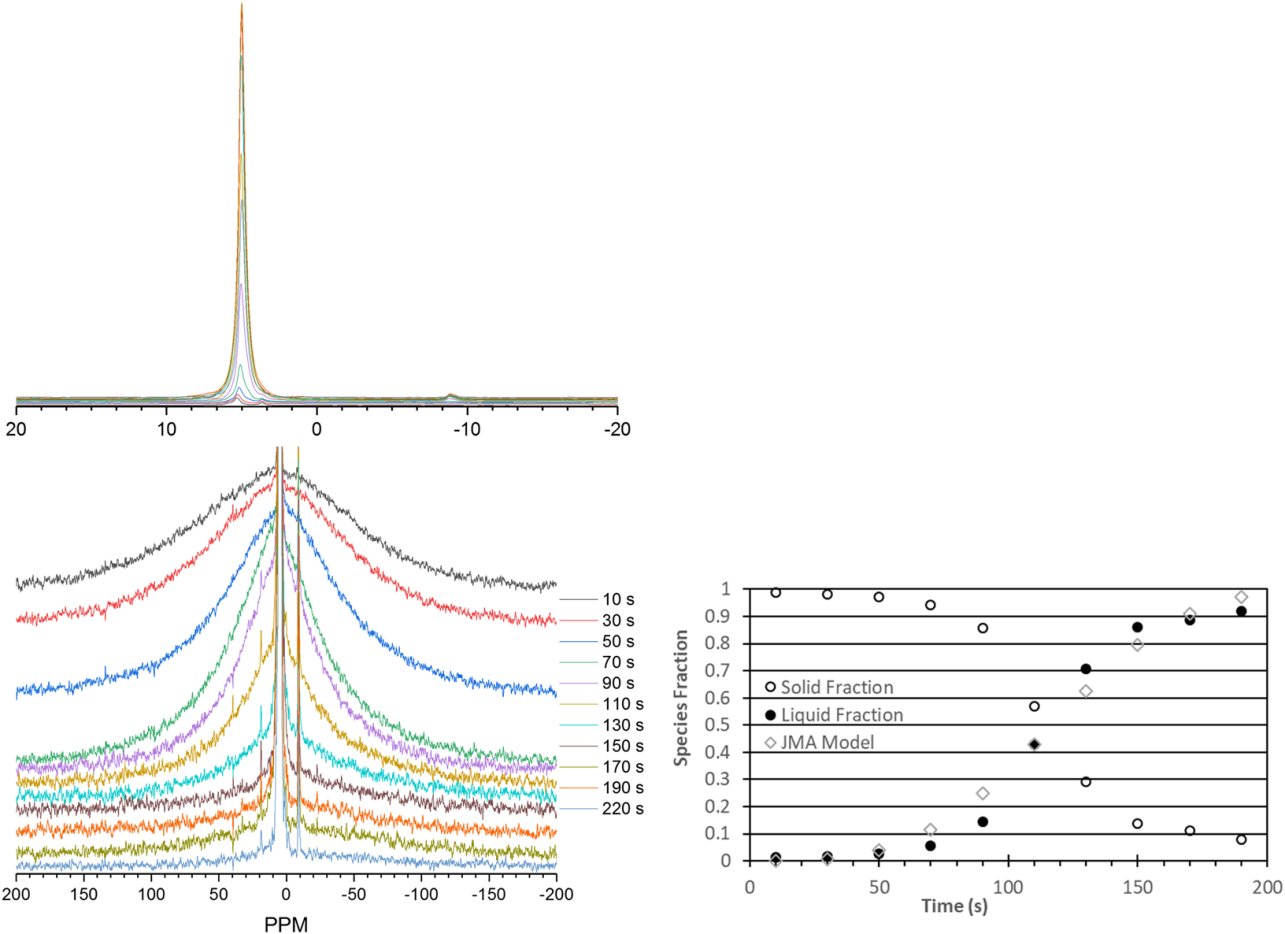
Transition from ice to liquid water of 200 μl of 20% H_2_^17^O where the liquid component (top left, zoomed view) is observed to increase as the broad solid feature (bottom left, expanded view) diminishes over the course of the phase transition. The graph on the right tracks the fraction of liquid and solid signal as a function of time during the experiment. A rough approximation with the Johnson-Mehl-Avrami model is presented as open diamonds.

At low temperatures, the water would freeze, dramatically reducing the spectral intensity of the liquid water in favor of a broad solid-state feature centered at ~7 ppm with a linewidth of ~30 kHz under MAS conditions. The linewidth of ice is known to be broad and the chemical shift to depend on the phase of ice^19,20^. MAS NMR was employed to narrow the linewidths of the solid and aid in the observation of the phase transition. Figure 3 illustrates the thawing process inside the rotor when the temperature was slightly elevated above the freezing point. The top series of spectra illustrate the increase in liquid signal as the ice melts. This line progressively intensifies over the course of the experiment at the expense of the solid ice (bottom series). Both species are plotted on the right as the fraction of water in a given state as a function of time, showing a smooth phase transition that mimics the profile of the Johnson-Mehl-Avrami-Kolmogorov model for phase transition. Disparities between the experimental data and model arise due to challenges in fitting the broad solid signal against the background and the non- isothermal conditions of the experiment. The solid signal linewidth narrows as the thawing processes occurs. The intensity of the solid component decreases over time concomitant with the liquid signal intensification.

**Figure 4:**
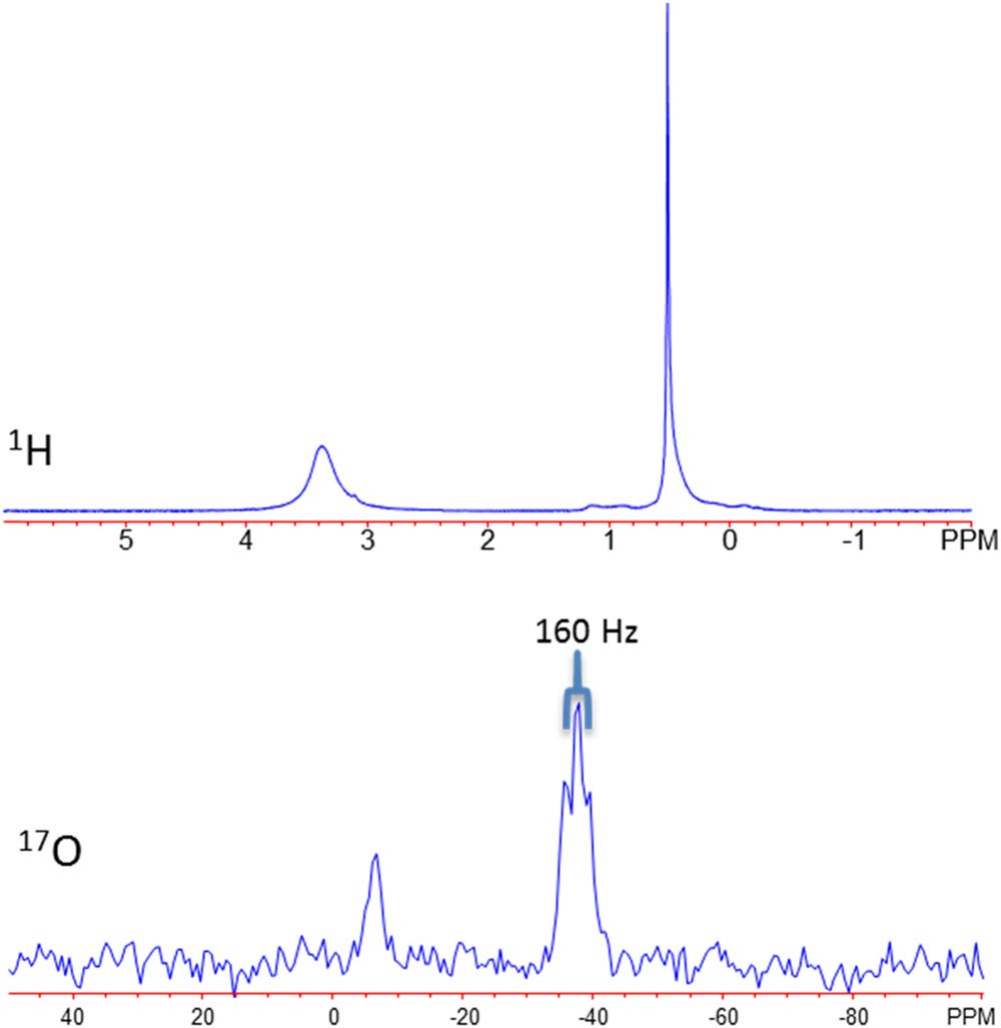
Representative ^1^H and ^17^O NMR spectra of 3.6 μl of water vaporized at 206 °C in the NMR rotor.

At elevated temperatures, the vapor pressure of water was enhanced [described by Antoine’s relationship as: $P{(T)}_{vap}\,(torr)={10}^{{C}_{0}-\frac{{C}_{1}}{T-{C}_{2}}}$ with C_0_: 8.14019, C_1_: 1810.91, and C_2_: 244.485] enabling the observation of the gas phase water signal^21^. Clear shielded shifts are observed in the gas phase chemical shifts, indicating substantially reduced interactions with neighboring water molecules and stronger covalent character. As shown in Fig. 4, the proton gas species resonate at ca. 0.5 ppm where the gaseous oxygen nuclei resonate at −37.9 ppm. The gas phase water ^1^H resonance can be modeled using simplified smaller DFT clusters (Table 1). At low water contents (<4 molecules) the molecules have reduced interactions with neighbors and the predicted chemical shifts (~0.1–0.6 ppm) are within the observed range for gas phase water. When additional water molecules are included into the cluster, a hydrogen bonding network is established and the prediction moves deshielded (~4.9 ppm). This theoretical consideration has been thoroughly modeled by DFT, statistical mechanics, and other computations to reflect enhanced accuracy and detail, showing the effect of entropy on chemical shielding^22–25^. ^1^H-^17^O spin-spin coupling (~80 Hz) is also observed for gaseous oxygen in water, indicating that a higher degree of charge transfer is present when the molecule is relatively isolated compared to the liquid state. This value is consistent with previous measurements^26,27^.

**Table 1:** Small DFT cluster model calculations of the ^1^H chemical shift in water as a function of cluster size.

Model	δ ppm ^1^H
TMS	0
1 H_2_O	0.06
2 H_2_O	0.40
3 H_2_O	0.58
4 H_2_O	4.39
5 H_2_O	4.3
6 H_2_O	4.2
7 H_2_O	4.9
8 H_2_O	4.3
9 H_2_O	5.1
10 H_2_O	5.0
12 H_2_O	4.9

**Figure 5:**
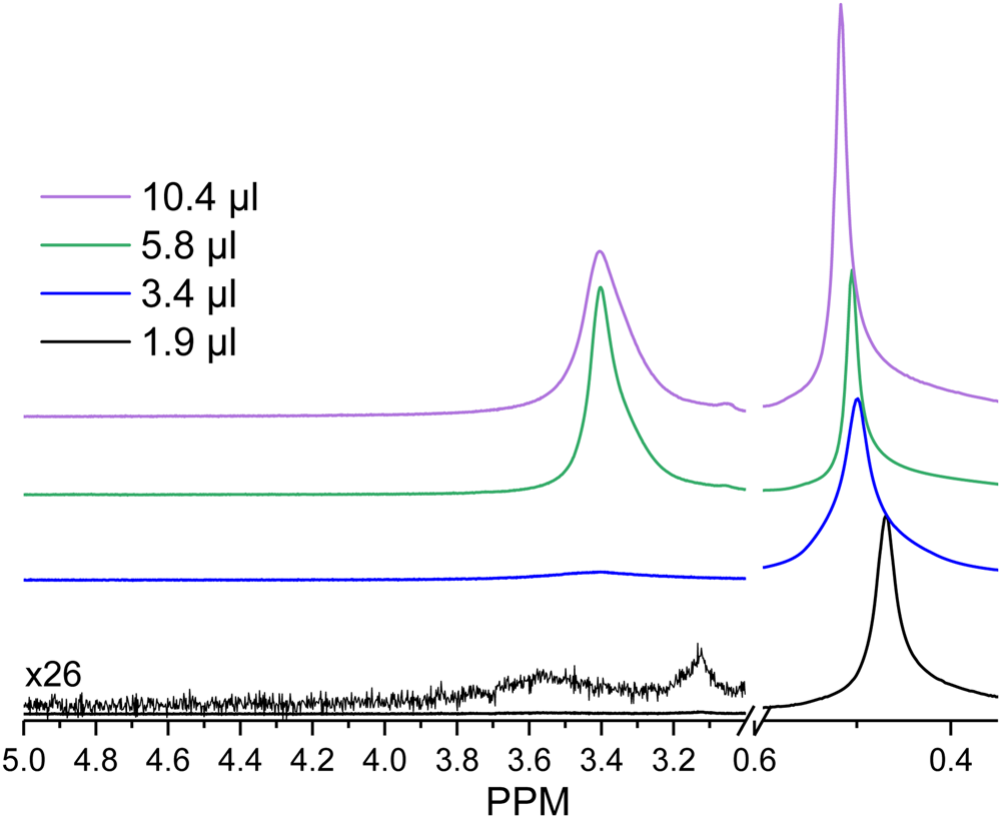
Gas to liquid exchange depicted by ^1^H NMR with differing degrees of gas fraction in the NMR rotor. Spectra were collected at 206 °C and 17.67 bar water vapor in a 294 μl NMR rotor.

The gas phase water chemical shifts are relatively independent of temperature compared to liquid phase water. In contrast, small differences are observed for contrasting gas:liquid fractions (Table 2 and Fig. 5). As the gas fraction increases, the gas phase ^1^H features deshield while the liquid phase peaks become more shielded, suggesting enhanced chemical exchange between the two phases as the surface area of the liquid phase increases under MAS conditions. This chemical exchange effect was minimized in Fig. 1 by reducing the void space in the rotor to decrease the surface are a and maintain an overwhelming majority of liquid state species. In systems with sufficient vapor fraction and interface however, this exchange effect may play an important role in the observed chemical shift of proton species and should be accounted for in the identification of species and interpretation of their mobility.

**Table 2:** Liquid and gas ^1^H chemical shifts at 206 °C and 17.67 bar water vapor at different gas to liquid fractions tuned by modulating the water content in a 294 μl rotor.

Water Content (uL)	^1^H PPM_Gas_	^1^H PPM_Liquid_	Theoretical Gas Fraction	Experimental Gas Fraction
1.9	0.47	3.54	1	0.96
3.4	0.50	3.42	0.69	0.67
5.8	0.51	3.40	0.40	0.26
10.4	0.52	3.40	0.22	0.15

**Figure 6:**
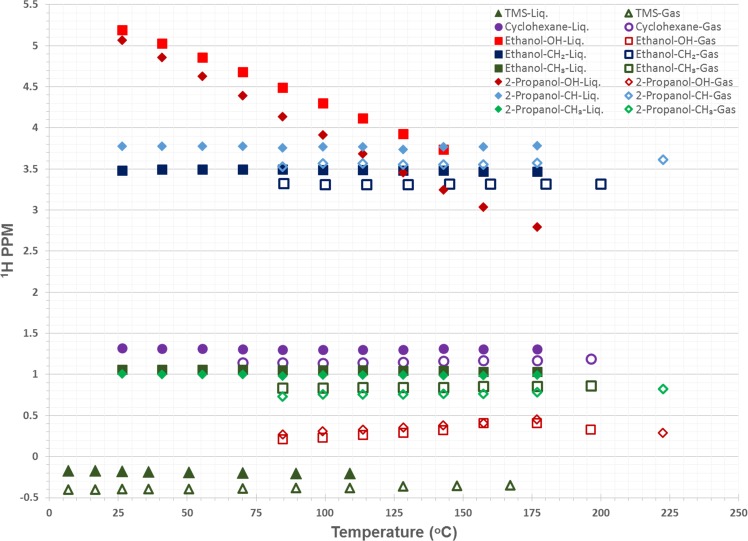
^1^H NMR chemical shift data for TMS, cyclohexane, ethanol, and isopropanol in the liquid and gas phase a function of temperature. Approximately 15 μl of liquid was added to the rotor during the runs to allow for the clear observation of gas phase constituents.

In addition to water, understanding the thermal behavior of other small molecules will be important as high-temperature, high-pressure, *in situ* NMR studies become increasingly common. While the relationships between temperature and methanol, ethylene glycol, or lead nitrate are well-documented (see experimental details), comparison to the thermal behavior of other representative molecules can provide general trends which may assist in understanding chemical shielding behavior in more complex systems. Figure 6 reports chemical shift data for each ^1^H environment in tetramethylsilane, cyclohexane, ethanol, and isopropanol. Clear trends are observed among the species present. The most obvious is relatively small changes in chemical shift with temperature of non-polar compounds TMS and cyclohexane due to their decreased interactions with neighboring molecules compared to water. Small decreases in the chemical shift of the liquid phase protons are observed for these two compounds, which may be indicative of chemical exchange with the gas phase present at higher temperatures. C_2_ and C_3_ alcohols, like methanol, show a temperature-dependent chemical shift. While the CH_3_ groups were relatively invariant with temperature due to their limited perturbation by bonding environment changes, protons on the primary carbon show limited increases in shielding at elevated temperatures, likely due to their proximity to the –OH group. The –OH group in these two alcohols shows a dramatic dependence on temperature. An increase in chemical shielding is present as the hydrogen bonding network is weakened. This effect is more pronounced in propanol, possibly due to weaker hydrogen bonds which are $A=\pi {r}^{2}$ more easily perturbed by thermal energy.

In all cases, the gas phase signals were shielded relative to their liquid phase counterparts. This effect was rather modest in non-polar compounds and methyl and methylene groups of the alcohols. Very slight increases in chemical shift of all gas-phase species were observed at higher temperatures, likely due to more rapid exchange with liquid-state species. This was most dramatic for -OH protons in the alcohols. The isopropanol gas peaks at ~225 °C were collected in the absence of liquid phase propanol, confirming the presence of chemical exchange between the two phases under conditions where both are present.

## Conclusion

Herein, we have summarized an extension of the known thermal perturbation of the properties of water and other small molecules of interest by MAS NMR and examined the trends of chemical shift, linewidth, and relaxation time from the perspective of the physical properties of these materials. The ^1^H and ^17^O NMR properties of chemical shift, linewidth, and spin-lattice relaxation time for water have been described over a wide range of temperatures with detailed thermal resolution. The findings highlight NMR evidence for the reduction of hydrogen bond strength at elevated temperature, which results in a decrease in chemical shift, narrowing of the linewidth, and decrease in correlation time. The ^1^H spectra of water highlight the formation of tetrahedral water cluster at lower temperatures and a distortion of this bonding order up to 50°C, evidenced by the ^1^H linewidth. The phase transitions of water between solid, liquid, and gas have been documented and summarized for water, and the ^1^H chemical shift properties in other small molecules have also been described over a range of temperatures, showing the limited effect of temperature on non-polar molecules, but dramatic effect on –OH groups involved in hydrogen bonding. Gas phase species are shielded relative to their liquid counterparts due to stronger covalent bonding in the absence of nearest-neighbor interactions. When significant gas phase species contact the liquid, chemical exchange plays an important role the observed chemical shifts for both liquid and gas species, which can obscure interpretation of the data if not accounted for in analysis. The results disclosed herein provide the foundation for understanding small molecules in more complex environments such as on a material surface or confined within a pore. Such understanding enables a clearer interpretation of complex systems during *in situ* NMR characterization at elevated temperatures and pressures.

## Methods

18.2 MΩ·cm water was generated by a Purelab Flex and mixed with H_2_^17^O water to formulate the 18.2 MΩ·cm 20% H_2_^17^O used in all experiments. Liquids were injected into the NMR rotor via microsyringe. All NMR spectra were collected with a 300 MHz Varian Inova NMR spectrometer operating at a ^1^H Larmor frequency of 299.97 MHz and a ^17^O Larmor frequency of 40.67 MHz. Samples were spun at the magic angle at a spinning rate of 4,000 Hz in a commercial 7.5 mm H-X ceramic probe. A 294 μl, high-temperature, high-pressure NMR rotor was employed to allow for system investigations at elevated temperatures and pressures^10–12^. ^1^H single pulse experiments were conducted with a pulse width of ~π/140 (0.1 μs), delay time of 200 ms, acquisition time of 1 s, and 32 repetitions. ^17^O single pulse experiments were conducted with a pulse width of π/2 (8 μs), delay time of 200 ms, acquisition time of 200 ms, and 128 repetitions. Inversion recovery experiments were conducted using a two-pulse sequence of π followed by π/2 (14 and 7 μs for ^1^H; 16 and 8 μs for ^17^O) the delay period was varied across 7 to 17 data points to collected the delay-modulated inversion recovery signal. A recycle delay of 10 s was used for ^1^H measurements and 200 ms for ^17^O; at least 4 repetitions were used in these measurements with similar acquisition times to the single pulse experiments. All ^1^H NMR data were externally referenced to adamantane at 1.82 ppm and ^17^O NMR data were externally referenced to 25°C water at 0 ppm. The collected inversion data were fit with y $(t)=\sum {A}_{i}\,(1-{B}_{i}{e}^{\frac{-t}{{T}_{1,i}}})$ where y(t) is the normalized spectral intensity as a function of delay time, A is the weighting factor for species i (sum to unity), B is the compensation factor for species i (1 < B ≤ 2, typically ~2), t is the time used in the pulse delay, and T_1_ is the spin-lattice relaxation time of species i. The experimental temperature was controlled using a commercial variable temperature heating stack. The heating stack flow rate was increased to dramatically reduce thermal gradients across the sample. Experimental temperatures were determined and verified with external calibrations employing ethylene glycol, lead nitrate, and methanol temperature thermometers which were repeatably correlated to the setting temperature of the heating stack at the employed stack flow rates and bearing and drive gas pressures^28,29^. All temperatures presented herein are based on the temperatures measured by external temperature calibration with the multi-nuclear NMR thermometers, not from the heating stack setting temperature. All free indication decays were processed without line broadening. Density functional theory calculations were carried out by the Amsterdam Density Functional (ADF) software^30–32^. Cluster model geometries were optimized using the generalized gradient approximation with Grimme’s third generation dispersion-corrections applied to the Beck-Lee-Yang-Parr functional (GGA: BLYP-D3)^33–35^. A Slater-type, all-electron, triple- ζ, two-polarization function (TZ2P) was used as the basis set^36^.
